# Distinct Profiles of Specialized Pro-resolving Lipid Mediators and Corresponding Receptor Gene Expression in Periodontal Inflammation

**DOI:** 10.3389/fimmu.2020.01307

**Published:** 2020-06-25

**Authors:** Brittney Ferguson, Nishantha R. Bokka, Krishna Rao Maddipati, Srinivas Ayilavarapu, Robin Weltman, Lisha Zhu, Wanqi Chen, W. Jim Zheng, Nikola Angelov, Thomas E. Van Dyke, Chun-Teh Lee

**Affiliations:** ^1^Department of Periodontics and Dental Hygiene, The University of Texas Health Science Center at Houston School of Dentistry, Houston, TX, United States; ^2^Department of Pathology, Wayne State University, Detroit, MI, United States; ^3^Bioinformatics and High Performance Computing Service Center, The University of Texas Health Science Center at Houston, Houston, TX, United States; ^4^Center for Clinical and Translational Research, The Forsyth Institute, Cambridge, MA, United States; ^5^Department of Oral Medicine, Infection, and Immunity, Faculty of Medicine, Harvard University, Boston, MA, United States

**Keywords:** inflammation, mass spectrometry, metabololipidomics, omega-3 fatty acids, omega-6 fatty acids, periodontitis, specialized pro-resolving lipid mediators

## Abstract

Polyunsaturated fatty acid-derived specialized pro-resolving lipid mediators (SPMs) play an important role in modulating inflammation. The aim of the study was to compare profiles of SPMs, SPM related lipid mediators and SPM receptor gene expression in gingiva of subjects with periodontitis to healthy controls. A total of 28 subjects were included; 13 periodontally healthy and 15 periodontitis before or after non-surgical periodontal therapy. Gingival tissues were collected from two representative posterior teeth prior to and 8 weeks after scaling and root planning; only once in the healthy group. Lipid mediator-SPM metabololipidomics was performed to identify metabolites in gingiva. qRT-PCR was performed to assess relative gene expression (2^−ΔΔCT^) of known SPM receptors. Intergroup comparisons were made using Wilcoxon tests. Thirty-six omega-6 or omega-3 fatty acid-derived lipid mediators and seven receptor genes were identified in gingiva. Profiles of lipid mediators and receptor gene expression were significantly different between the three groups. Levels of six lipid mediators, 5-HETE, 15-HETE, 15(S)-HEPE, 4-HDHA, 7-HDHA, and 17-HDHA in periodontitis before treatment were significantly higher than in periodontitis after treatment. The expression of *BLT1* in the healthy group was significantly higher than periodontitis subjects before and after treatment. The expression of *GPR18* in periodontitis before treatment was significantly higher than in periodontitis after treatment while the expression of *GPR32* in periodontitis before treatment was significantly lower than in periodontitis after treatment. Elevated levels of SPM biosynthetic pathway markers in periodontitis subjects before treatment indicated inflammation induced pro-resolution activity in gingiva, but receptors for these molecules were deficient in periodontitis pre-treatment suggesting that failure of resolution of inflammation contributes to excess, chronic inflammation in periodontitis.

## Introduction

Periodontitis is a biofilm-induced chronic inflammatory disease that is characterized by gingival inflammation, loss of connective tissue attachment and alveolar bone, which if severe enough, can potentially lead to tooth loss. In the United States, around 64 million adults have periodontitis (46%) with 8.9% having severe periodontitis (1). The global costs of lost productivity from severe periodontitis alone have been estimated to be 54 billion US Dollars every year (2). The disproportionate host response and dysbiosis of the oral microbiome are the two major etiologic factors in the pathogenesis of periodontitis (3). During pathogenesis, subjects initially develop an acute inflammatory response in the gingiva characterized by the presence of mostly neutrophils and macrophages. Persistence of inflammation leads to the development of a chronic lesion characterized by the predominance of plasma cells. The continued inflammation induced by concomitant stimulation by bacteria and their by-products triggers the host response to mediate irreversible destruction of the affected periodontal tissues (3, 4).

Specialized pro-resolving lipid mediators (SPMs), including lipoxins, resolvins, protectins, and maresins, are derived from essential fatty acids (omega-6 and omega-3 fatty acids) that have dual actions: anti-inflammation and resolution of inflammation. In the phase of inflammation resolution, SPMs bind to their corresponding G protein-coupled receptors on cells signaling reduction of excessive neutrophil recruitment to the site, as well as promotion of neutrophil apoptosis and removal by non-phlogistic macrophage efferocytosis (5). The imbalance between the levels of pro-resolution mediators and pro-inflammatory mediators has been linked to several chronic inflammatory diseases, including periodontitis, atherosclerosis, mastitis, asthma, cystic fibrosis, and others (6–9).

In periodontitis, pro-inflammatory lipid mediators are known to increase dramatically in periodontal tissues and gingival crevicular fluid (GCF) (10–12). Early response lipid mediators, leukotriene B_4_ (LTB_4_) and prostaglandin E_2_ (PGE_2_), are markedly increased (13). PGE_2_ is elevated in GCF of people with periodontitis and the increases correlate well with disease severity and ongoing activity (13, 14). Neutrophils from people with periodontitis produce increased lipoxin A_4_ (LXA_4_), one of SPMs, compared to subjects without periodontal disease (15). LXA_4_ upregulation and *in vivo* neutrophil priming are also observed in asthma patients (16).

Localized Aggressive Periodontitis (LAP) is a rapidly progressing form of periodontitis characterized by compromised phagocytic ability of neutrophils and macrophages (17, 18) that seemed refractory to endogenous levels of lipoxins (15), but could be rescued by other SPMs. Dysregulation of resolution in LAP was attributed to aberrant lipoxygenase activity demonstrated in whole blood. Additionally, surface P-selectin expression on LAP platelets, CD18 expression on circulating neutrophils and monocytes were increased, which resulted in significantly greater platelet-neutrophil and platelet-monocytes aggregates in circulating whole blood (17). Taken together, these observations suggest that failure to resolve local inflammatory insults induced by bacteria in the periodontium leads to periodontal disease progression. Interestingly, the *in vitro* abnormalities in LAP were all reversed with addition of resolvin E1 (RvE1). Hasturk and co-workers (19) reported that LAP neutrophils respond to resolvins, but not lipoxins. These findings bring up the interesting prospect of selective abnormalities of the response to individual pro-resolving mediators based on specific ligand-receptor interactions.

Several pre-clinical studies have demonstrated that treatment with exogenous SPMs effectively prevents destruction and helps regeneration of lost tissues in experimental periodontitis (20, 21). Additionally, SPMs were identified in oral fluids (8, 15) and clinical studies have highlighted the associations between SPM levels in gingival crevicular fluid, saliva, or serum and inflammatory conditions in subjects with aggressive or chronic periodontitis (8, 22). These results support the concept that the failure of resolution of an acute active inflammatory process results in periodontitis. We hypothesize that the relative levels of SPMs and SPM pathway markers regulating inflammation resolution in periodontal tissues play an important role in periodontal inflammation. However, levels of these lipid mediators and expression of their corresponding receptor genes have not been assessed directly in gingiva, the site of the inflammation.

At this time, the relationship between pro-resolution mediators in gingiva and periodontitis is largely unknown. The aim of this study was to profile SPMs and SPM related lipid mediators (LMs), as well as to determine SPM receptor gene expression in gingiva in periodontally healthy and diseased subjects. The profiles of SPM related lipid mediators and their receptor gene expression were found to be associated with periodontal inflammatory status and were modified by periodontal treatment. The results of this study further clarify the biological role of SPMs in periodontitis and its application to periodontal diagnosis and therapy.

## Materials and Methods

### Clinical Study Design

The study was conducted in accordance with the guidelines of the World Medical Association's Declaration of Helsinki and approved by the University of Texas Health Science Center at Houston (UTHealth) Committee for the Protection of Human Subjects (HSC-DB-16-0167). All participants provided written informed consent.

Inclusion criteria: subjects aged 18 to 75 years of age with ≥24 teeth and no history of systematic periodontal therapy within the past 2 years. All subjects should not have received systemic antibiotics or anti-inflammatory drugs for ≥3 months within the past year, did not routinely take fish oil supplements, had no presence of diabetes mellitus or any systemic condition that entails a diagnosis of “systemic disorders that have a major impact on the loss of periodontal tissues by influencing periodontal inflammation,” were not pregnant, and not current users of tobacco products or nicotine replacement medication.

The subjects in the periodontally healthy group were required to have all teeth with a probing depth of ≤3 mm, clinical attachment loss of ≤2 mm (except teeth with mid-buccal or lingual gingival recession), and radiographic bone levels ≤2 mm from the cementoenamel junction (CEJ). The subjects in the periodontitis group were required to present with ≥8 teeth with a probing depth of ≥5 mm, clinical attachment loss of ≥3 mm, and radiographic bone levels >2 mm from the CEJ. Subjects were questioned on their history and extent of periodontal treatment, which was documented to confirm inclusion in the study.

### Clinical Evaluation

A baseline pre-experimental evaluation was performed for all subjects, which included clinical and radiographic assessment of the oral cavity. Probing depth was measured in millimeters at 6 sites per tooth as determined by a UNC-15 periodontal probe. Bleeding on probing (BOP) was assessed after probing, utilizing a dichotomous scoring system of (1) and (0), equating to presence and absence of BOP, respectively. The percentage of sites with BOP was calculated. Measurement of the distance from the free gingival margin (FGM) to the CEJ (FGM-CEJ) was recorded with the periodontal probe. Clinical attachment level (CAL) was calculated by subtracting the FGM-CEJ measurement from the probing depth.

Full mouth series (FMS) radiographs were obtained prior to all treatments for all subjects in the UTHealth School of Dentistry (UTSD) clinics. Therefore, no additional radiographs were taken for purposes of the study, unless the FMS radiographs were taken ≥3 years prior or progression of periodontal disease was noted. Evaluation of the FMS radiographs of all teeth measuring the distance between the CEJ and alveolar bone was performed using a calibrated measurement tool (MiPACS, Charlotte, NC, USA) to confirm periodontal status.

### Clinical Sample Collection

Subjects in the healthy (H) group were seen for one clinical research visit, prior to prophylaxis. During the visit, four biopsies of gingival tissues (papilla) were obtained from interproximal sites of 2 representative posterior teeth (mesiobuccal, distobuccal, mesiolingual, or distolingual sites of each tooth). Subjects in the periodontitis group were seen for two clinical visits; the first visit being prior to the first scaling and root planning (SRP) appointment (the *P* group), and the second visit 8–10 weeks following completion of the second SRP appointment (the A group). During each visit, four biopsies of gingival tissue were collected from interproximal sites of 2 representative posterior teeth. The most severely affected teeth with more bone loss than other teeth were selected for gingival sample collection. All first-visit collected gingival tissues in the periodontitis subjects were from sites with deep probing depths (≥5 mm). The second visit also included full mouth periodontal charting and re-evaluation prior to gingival sample collection. All gingival samples were collected from the same sites as the first visit.

At each site for gingival sample collection, topical benzocaine was applied followed by local anesthetic (4% Septocaine with 1:100,000 epinephrine) infiltration buccally and lingually. Care was taken to not directly inject into the papillary regions to prevent introduction of local anesthetic in the gingival tissues. Gingival samples were collected from interproximal sites of the tooth using either a 15c or 12D blade. Intrasulcular incisions were made from the papilla zenith toward the base of the interproximal papillae, but not to exceed the nearest line angle of each tooth. A subsequent horizontal incision was made at the base of the interproximal papillae to connect the intrasulcular incisions. The incisions extended to the alveolar bone, and split thickness papillae were partially elevated to permit the harvesting of all the interproximal papillae. The gingival sample was required to include epithelium and the underlying supracrestal connective tissue. One of the gingival samples was placed in RNAlater^TM^ (Thermo Fisher Scientific, Waltham, MA, USA) for real-time quantitative reverse transcription (qRT-PCR) assay and stored in a −80°C freezer. The remaining three gingival tissue samples were collected in storage vials placed in liquid nitrogen and subsequently transferred to a −80°C freezer until time of analysis. One of the three snap-frozen gingival samples from each subject was selected for LM-SPM metabololipidomic analysis. In the four gingival samples, the sample selected for LM-SPM metabololipidomic analysis was from sites with the deepest probing depth and the sample selected for qRT-PCR assay was from sites with the second-deepest probing depth.

### LM-SPM Metabololipidomics for Gingival Samples

The gingival samples were quantitatively analyzed for levels of SPMs and other LMs using LM-SPM metabololipidomics as described earlier with the following modifications (23).

Gingival samples were homogenized using zirconium beads (Precellys homogenizer) before extraction and analysis by liquid chromatography with tandem mass spectrometry (LC-MS/MS) analysis. Protein concentration (measured by BCA method) in the homogenate was used for normalization of the LM data. LC-MS/MS grade methanol was added to each sample to a final concentration of 15% along with internal standards (5 ng each of PGE1-d4, RvD2-d5, LTB4-d4, and 15-HETE-d8). The samples were sonicated in a bath sonicator for 2 min and left on ice for 1 h in the dark. The samples were applied to pre-conditioned C18 solid phase extraction cartridges (StrataX C18, 30 mg, Phenomenex, conditioned with 2 ml methanol followed by 2 ml water containing 15% methanol), washed with 2 ml 15% methanol in water followed by 2 ml hexane, and dried under vacuum. The cartridges were eluted directly into high performance liquid chromatography (HPLC) autosampler vials with 1 ml methanol containing 0.1% formic acid. The eluates were evaporated to dryness under a gentle stream of nitrogen while maintaining the external temperature at 25°C. The dried residue was immediately reconstituted in methanol, vials flushed with nitrogen, capped, and stored at −80°C until analysis. At the time of LC-MS/MS analysis, the samples were thawed to room temperature, and equal volume of 25 mM aqueous ammonium acetate was added, vortex mixed, and loaded in the autosampler maintained at 15°C. HPLC was performed on a Prominence XR system (Shimadzu) using Luna C18 (3 μl, 2.1 × 150 mm) column. The mobile phase consisted of a gradient between A: methanol-water-acetonitrile (10:85:5 v/v) and B: methanol-water-acetonitrile (90:5:5 v/v), both containing 0.1% ammonium acetate. The gradient program with respect to the composition of B is as follows: 0–1 min, 50%; 1–8 min, 50–80%, 8–15 min, 80–95%; and 15–17 min, 95%. The flow rate was 0.2 ml/min. The HPLC eluate was directly introduced to ESI source of QTRAP5500 mass analyzer (SCIEX) in the negative ion mode with the following conditions: Curtain gas, GS1, and GS2: 35psi, Temperature 600°C, Ion Spray Voltage:−2500 V, Collision gas: low, and Declustering Potential:−60 V. The MRM was scheduled to monitor each transition for 120 s around the established retention time for each LM. Optimized Collisional Energies (18–35 eV) and Collision Cell Exit Potentials (7–10 V) were used for each MRM transition. Mass spectra for each detected LM were recorded using the Enhanced Product Ion (EPI) feature to verify the identity of the detected peak in addition to MRM transition and retention time match with the standard. The data were collected using Analyst 1.7 software and the MRM transition chromatograms are quantitated by MultiQuant software (both from SCIEX). The internal standard signals in each chromatogram were used for normalization and recovery as well as relative quantitation of each analyte. The quantities of SPMs and other LMs in gingival samples are presented as mean ± standard deviation ng per mg of the total protein in each sample. The lower limit of quantitation is 0.015 ng.

### Expression of SPM Receptor Genes via qRT-PCR

Total RNA was extracted from the gingival tissue utilizing a RNeasy Fibrous Tissue Mini kit (QIAGEN Inc., Valencia, CA) as per the manufacturer's protocol. First, the gingival tissue was disrupted and homogenized with a glass homogenizer containing 300 μl of kit buffer RLT mixed with β-Mercaptoethanol; 10 μl of Proteinase K diluted in 590 μl of RNase-free water was added, followed by ethanol. The sample was washed in a spin column followed by a rinse with 350 μl of kit buffer RW1. DNase 1 (10 μl) stock solution was mixed with 70 μl of kit buffer RDD and applied to the spin column, followed by another 350 μl of kit buffer RW1. The spin column was rinsed with 500 μl of kit buffer RPE 2 times, followed by a final administration of 30 μl of nuclease-free water, which was collected in a 1.5 ml collection tube. The concentration and purity of each RNA extraction sample was measured by the A_260_/A_280_ ratio spectrophotometrically (NanoDrop 2000c, Thermo Fisher Scientific, Waltham, MA, USA).

The extracted RNA was reverse transcribed to complimentary DNA (cDNA) and used for qRT-PCR. The RNA concentration in each sample was calculated to determine the volume required for 1 μg of RNA per sample. RNase-free water was then added to make a total volume of 10 μl. A reverse transcription master mix was prepared consisting of dNTP, buffer, primers, nuclease-free water, reverse transcriptase, and RNase inhibitor (High Capacity cDNA Reverse Transcription Kit, Applied Biosystems, Grand Island, NY). 10 μl of the master mix was added to each RNA sample tube and placed in the thermocycler for reverse transcription at 25°C for 10 min, 37°C for 120 min, 85°C for 5 min, and then 10°C in a thermal cycler (3Prime, Techne, Staffordshire, UK). The cDNA was stored at 4°C short term (<24 h) and at−20°C long term.

The cDNA was analyzed for SPM receptor gene expression with qRT-PCR, utilizing the TaqMan Gene Expression Assays Protocol (Applied Biosystems, Foster City, CA, USA). A PCR master mix (18 μl) was prepared consisting of TaqMan Fast Advanced Master Mix (10 μl), TaqMan Assay primers (1 μl), and nuclease-free water (7 μl). The TaqMan Assay primers included *GAPDH* (assay ID: Hs99999905_m1, Applied Biosystems) as the housekeeping gene compared to *ALX (FPR2)* (assay ID: Hs00265954_m1, Applied Biosystems), *BLT1 (LTB4R)* (assay ID: Hs00175124_m1, Applied Biosystems), *ChemR23 (ERV1)* (assay ID: Hs01386063_m1, Applied Biosystems)*, GPR18 (DRV2)* (assay ID: Hs00245542_m1, Applied Biosystems)*, GPR32 (DRV1)* (assay ID: Hs00265986_s1, Applied Biosystems)*, GPR37* (assay ID: Hs0017374_m1, Applied Biosystems), and *LGR6* (assay ID: Hs00663887_m1, Applied Biosystems), which were labeled with FAM dye. Each receptor binds to particular SPMs, which can clarify whether the SPMs present in gingiva have corresponding receptors for binding to cells to stimulate pro-resolving activity (Table 1). cDNA template (2 μl) was added to the PCR reaction mix (18 μl) in each well of a 96-well optical reaction plate. Each cDNA sample was tested in triplicate for each primer to verify the readings. The plate was sealed with optical adhesive film and centrifuged at 3,000 RPM for 3 min to bring the PCR reaction mix to the bottom of the wells. qRT-PCR was performed for each plate in an automated thermal cycler (StepOnePlus™ System, Applied Biosystems, Foster City, CA, USA) at 50°C for 2 min (one cycle), 95°C for 20 s, 95°C for 1 s, and 60°C for 20 s (40 cycles total). The calculated cycle threshold (Ct) values from each sample were obtained and analyzed through a software interface and spreadsheet for the calculation of relative expression (2^−ΔΔCt^) to determine expression of specific SPM receptor genes between groups. The *GAPDH* gene was the housekeeping gene and one of the samples in the healthy group was selected as the control sample. The Ct values for the target gene in the tested sample, target gene in the control sample, housekeeping gene in the test sample and housekeeping gene in the control sample were used to calculate ΔΔCt.

**Table 1:** Corresponding receptors of specialized pro-resolving lipid mediators (SPMs).

**Genes of SPM receptors**	**SPMs**	**References supporting corresponding SPMs**
*ALX (FPR2)*	LXA4 RvD1 RvD3	Lipoxins (24); D-series resolvins (25, 26)
*BLT1 (LTB4R)*	RvE1 RvE2	E-series resolvins (27, 28)
*ChemR23 (ERV1)*	RvE1 RvE2	E-series resolvins (27, 28)
*GPR18 (DRV2)*	RvD2	RvD2 (29)
*GPR32 (DRV1)*	RvD1 RvD3 RvD5	D-series resolvins (26, 30, 31)
*GPR37*	PD1	PD1 (32)
*LGR6*	MaR1	MaR1 (33)

*Genes of receptors to which specific SPMs can bind are listed*.

### Statistical Analysis

Demographic and clinical data were analyzed using Chi-squared test, student's *t*-test or paired *t*-test to evaluate group balance of variables. The LM-SPM metabololipidomics data and gene expression data were first log transformed, and Wilcoxon rank sum test was used to detect differentially expressed LMs and genes between two conditions except for comparison between groups P and A, where Wilcoxon signed rank test was applied. The *p*-values were adjusted using False Discovery Rate (FDR) multi-test correction method to reduce statistical significance by chance. Linear mixed-effects regression analysis was applied to assess the effects of different variables on levels of lipids and gene expression. If the target (lipid or gene) had a *p*-value < 0.1 in linear mixed-effects regression, the comparisons between groups were further adjusted for confounders. Pairwise comparisons between groups were performed using Tukey's test. Principal Component Analysis (PCA) was used to demonstrate the profiles of lipid mediator and receptor gene expression data. Analysis of Similarities (ANOSIM) was used to compare lipid mediator profiles or receptor gene expression profiles between groups. Data were considered statistically significant when *p*-value < 0.05. All analyses were performed in R software 3.6.2.

## Results

According to a power analysis of preliminary results of LM-SPM metabololipidomics, at least 12 subjects per group were needed to detect a difference in the level of 14-HDHA, the maresin pathway marker, with 1.27 effect size and 80% power. A total of 30 subjects, 15 subjects in the periodontitis groups and 15 subjects in the healthy group, were recruited on the basis of their periodontal status. Two subjects from the healthy group were disqualified from the study when it was found that the subjects were current smokers, leaving a total of 28 subjects qualified for the study. According to the 2017 World Workshop on the Classification of Periodontal and Peri-Implant Diseases and Conditions, all periodontitis subjects were diagnosed with generalized periodontitis, Stage II or III, Grade B or C. The mean age in the healthy subjects was significantly lower than in the periodontitis subjects. The mean probing depths of sample collection sites in the healthy group were significantly lower than in the periodontitis prior to and after SRP groups (P and A groups) (Table 2).

**Table 2:** Characteristics of subjects and sample collection sites.

	**Healthy (H)**	**Periodontitis before non-surgical therapy (P)**	**Periodontitis after non-surgical therapy (A)**	***p*-value (H vs. P/A)**	***p*-value (P vs. A)**
Age	39.31 ± 15.80	51.20 ± 9.99	51.20 ± 9.99	0.02/0.02	NA
Gender (Male/Female)	6/7	8/7	8/7	0.71/0.71	NA
PD (mm) of sites for metabololipidomics	2.85 ± 0.38	6.47 ± 1.13	5.33 ± 1.76	<0.01/<0.01	0.01
PD (mm) of sites for qRT-PCR	2.85 ± 0.38	5.87 ± 1.13	4.13 ± 1.51	<0.01/0.01	<0.01

*PD, probing depth; NA, not available due to the subjects are the same in the P and A groups; p-values were calculated using Student's t-test, paired t-test or Chi-squared test*.

### Distinct Profiles of Lipid Mediators and Receptor Gene Expression

**Figure 1:**
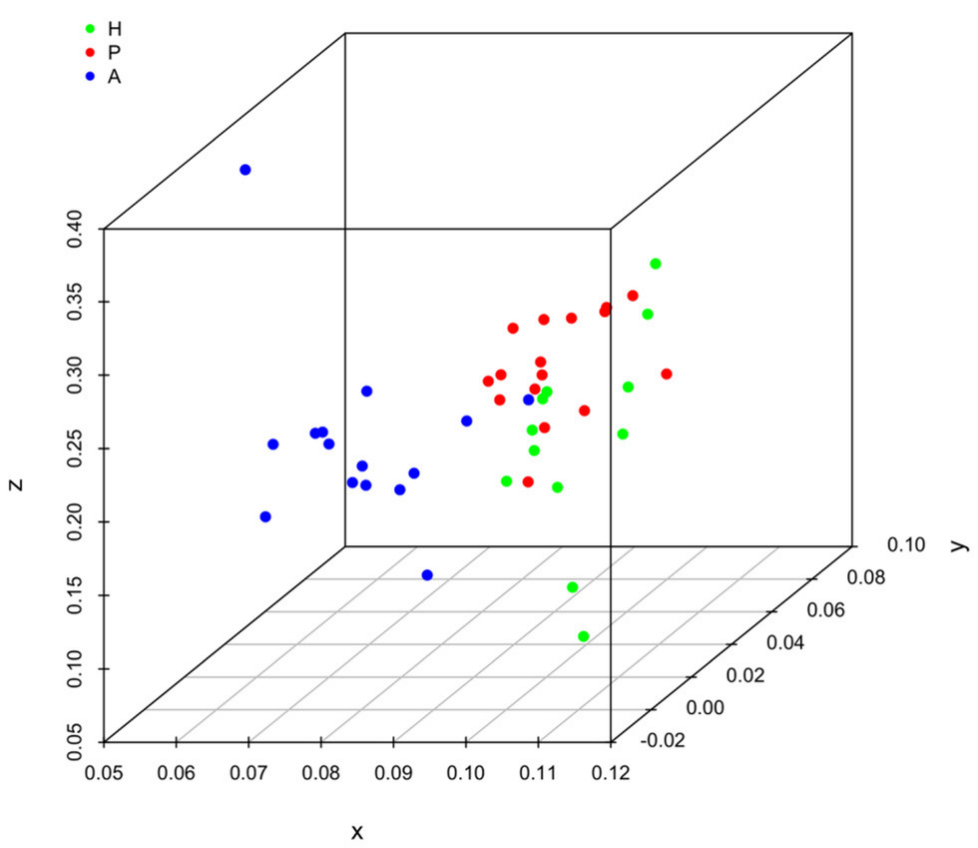
Principal component analysis (PCA) plot for profiles of specialized pro-resolving lipid mediators (SPMs) and lipid mediators (LMs) in human gingival. One dot represents one sample in each group. This plot demonstrates clusters of samples based on their similarity of lipid mediator levels. (H (green): healthy; P (red): periodontitis before non-surgical therapy; A (blue): periodontitis after non-surgical therapy).

**Figure 2:**
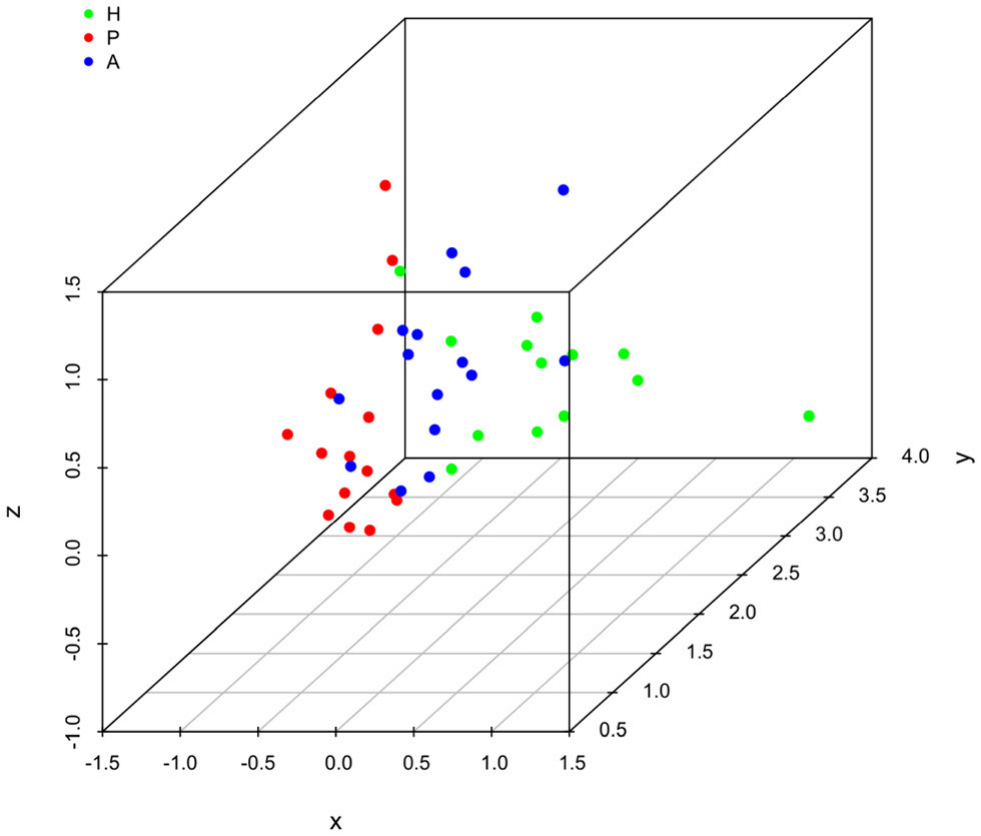
Principal component analysis (PCA) plot for profiles of specialized pro-resolving lipid mediator (SPM) receptor gene expression in human gingiva. One dot represents one sample in each group. This plot demonstrates clusters of samples based on their similarity of receptor gene expression. (H (green): healthy; P (red): periodontitis before non-surgical therapy; A (blue): periodontitis after non-surgical therapy).

It is clear that lipid mediator profiles in gingiva were markedly different between patients with periodontitis prior to SRP, after SRP and healthy controls by distinct clustering of samples from different inflammation severity groups (Figure 1). Similarly, distinct clustering of samples between the three groups were also present in receptor gene expression. The response to non-surgical periodontal treatment was a return to gene expression consistent with healthy subjects (Figure 2).

Results of ANOSIM showed both lipid mediator profiles and SPM receptor gene expression profiles were significantly different between the three groups (*p* < 0.01, 0.02, respectively). In the *post hoc* analysis, the lipid mediator profile in periodontitis prior to SRP (the P group) was significantly different from the profile in periodontitis after SRP (the A group) (*p* < 0.01). The receptor gene expression profiles in periodontitis prior to SRP (the P group) and after SRP (the A group) were both significantly different from the profiles in health controls (the H group) (*p* = 0.01, 0.046, respectively). These results are consistent with the distinct clustering of samples between the three groups in PCA plots (Figures 1, 2).

### Levels of SPMs and LMs in Gingival Tissues

After assessing the lipid mediator profiles (Figure 1), we wanted to know whether levels of specific lipid mediators were different between the three groups. These lipid mediators potentially could be biomarkers indicating the status of periodontal inflammation. Also, these results may elucidate the role of these lipid mediators in periodontitis. A total of 50 omega-3 or omega-6 fatty acid-derived lipid mediators were measured from 1 representative gingival sample of each study subject. In the healthy and periodontitis prior to SRP groups, 36 out of 50 lipids were detected, while 35 of 50 lipids were detected in the periodontitis after SRP group. Varying levels of SPM pathway markers including 5-HETE, 5(S),12(S)-DiHETE, 12-HETE, 12(S)-HHTrE, 15-HETE, 11-HEPE, 12-HEPE, 18-HEPE, 4-HDHA, 17-HpDHA, 14-HDHA, PGE2, PGF2a, and TXB2 were identified in all samples. Varying levels of SPMs including LXA4, RvE3, RvD1, RvD5, RvD6, PD1, PD1 (n-3, DPA), maresin 1 (MaR1), and 7(S)-Maresin 1 (S-epimer for MaR1), were detected in all three groups, but not in every sample (Table 3).

**Table 3:** Levels of lipid mediators in gingival tissues (ng/mg total protein).

**SPMs and LMs**	**Healthy (H)** ***n* = 13**	**Detection frequency**	**Periodontitis before non-surgical therapy (P)** ***n* = 15**	**Detection frequency**	**Periodontitis after non-surgical therapy (A)** ***n* = 15**	**Detection frequency**
LXB4	0	0	0	0	0	0
LXA5	0	0	0	0	0	0
LXA4	0.03 ± 0.02	4	0.02 ± 0.01	9	0.01 ± 0.00	1
15-epi LXA4	0	0	0	0	0	0
**5-HETE**	0.97 ± 0.59	13	**2.05 ± 1.69**	**15**	**0.7 ± 0.38**	**15**
5(S),6(R)-DiHETE	1.98 ± 0.99	13	2.69 ± 2.41	15	4.69 ± 3.93	10
5(S),12(S)-DiHETE	18.71 ± 25.78	13	13.46 ± 11.22	15	20.88 ± 21.31	15
5(S),15(S)-DiHETE	0.06 ± 0.04	2	0.08 ± 0.04	7	0.05 ± 0.03	2
11-HETE	1.69 ± 0.73	13	2.06 ± 0.83	15	1.49 ± 0.45	14
12-HETE	108.10 ± 48.66	13	137.32 ± 64.98	15	102.97 ± 49.5	15
12(S)-HHTrE	1.14 ± 0.75	13	1.08 ± 0.57	15	1.00 ± 0.43	15
**15-HETE**	2.94 ± 1.43	13	**5.57 ± 3.11**	**15**	**2.45 ± 0.96**	**15**
RvE1	0	0	0	0	0	0
RvE2	0	0	0.01 ± 0.01	3	0.01 ± 0.00	1
RvE3	0.04 ± 0.02	6	0.07 ± 0.05	11	0.12 ± 0.16	10
5-HEPE	0.06 ± 0.03	6	0.08 ± 0.06	12	0.04 ± 0.02	6
5(S),15(S)-DiHEPE	0.11 ± 0	1	0	0	0.16 ± 0.14	2
11-HEPE	0.08 ± 0.06	13	0.07 ± 0.03	15	0.04 ± 0.02	15
12-HEPE	10.08 ± 7.56	13	10.04 ± 5.57	15	6.60 ± 4.14	15
**15(S)-HEPE**	0.14 ± 0.08	8	**0.18 ± 0.09**	**14**	**0.08 ± 0.04**	**13**
18-HEPE	0.06 ± 0.04	13	0.05 ± 0.03	15	0.04 ± 0.02	15
RvD1	0.09 ± 0.13	9	0.02 ± 0.02	9	0.05 ± 0.05	11
AT-RvD1	0.01 ± 0	1	0	0	0	0
RvD2	0	0	0	0	0	0
RvD3	0	0	0	0	0	0
AT-RvD3	0	0	0	0	0	0
RvD4	0	0	0	0	0	0
RvD5	0.03 ± 0.01	2	0.02 ± 0.01	8	0.01 ± 0.01	3
RvD5(n-3, DPA)	0	0	0	0	0	0
RvD6	0.07 ± 0.03	6	0.09 ± 0.08	7	0.16 ± 0.18	5
**4-HDHA**	0.03 ± 0.01	13	**0.03 ± 0.02**	**15**	**0.02 ± 0.01**	**15**
**7-HDHA**	0.03 ± 0.01	12	**0.04 ± 0.02**	**15**	**0.02 ± 0.01**	**14**
13-HDHA	0.23 ± 0.14	13	0.23 ± 0.14	15	0.16 ± 0.1	13
PD1	0.025 ± 0.00	2	0.02 ± 0.01	6	0.03 ± 0.00	3
PD1 (n-3,DPA)	0.08 ± 0.02	2	0.06 ± 0.05	2	0.10 ± 0.08	3
AT-PD1	0.00 ± 0.00	1	0.02 ± 0.00	2	0	0
22-OH-PD1	0	0	0	0	0	0
10S,17S-DiHDHA	0	0	0	0	0	0
**17-HDHA**	0.15 ± 0.13	13	**0.26 ± 0.2**	**15**	**0.11 ± 0.06**	**15**
Maresin1	0.12 ± 0.11	13	0.10 ± 0.08	15	0.12 ± 0.11	15
7(S)-Maresin1	0.03 ± 0.03	6	0.03 ± 0.03	13	0.02 ± 0.01	7
Maresin 1 (n-3, DPA)	0.038 ± 0.04	3	0.01 ± 0.00	1	0	0
14-HDHA	4.76 ± 3.26	13	4.71 ± 2.47	15	3.72 ± 2.25	15
LTB4	3.42 ± 5.16	10	1.24 ± 1.04	13	1.96 ± 2.17	10
20-OH-LTB4	0	0	0.01 ± 0.01	5	0.00 ± 0.00	4
20-COOH_LTB4	0	0	0	0	0	0
PGD2	4.05 ± 3.6	9	3.5 ± 2.63	13	3.25 ± 4.14	14
PGE2	33.12 ± 29.67	13	27.2 ± 23.82	15	20.52 + 14.48	15
PGF2a	3.82 ± 3.26	13	2.64 ± 2.11	15	2.89 ± 2.41	15
TXB2	3.39 ± 2.41	13	4.50 ± 2.59	15	3.48 ± 1.39	15

*Lipid mediators highlighted in bold indicate statistically significant difference (FDR adjusted p-value < 0.05). Detection frequency indicates how many times the target was detected in samples of the group. Lipid mediators (SPMs and pathway markers) are grouped by biosynthetic pathways: Lipoxins (derived from omega-6 arachidonic acid), orange; E-series Resolvins (derived from omega-3 EPA), yellow; D-series Resolvins (derived from omega-3 DHA or DPA), green; Protectins (derived from omega-3 DHA or DPA), blue; Maresins (derived from omega-3 DHA or DPA), red; pro-inflammatory lipid mediators, purple. SPMs are listed first in each group; followed by their pathway markers. Some pathway markers are involved in multiple pathways but only one major pathway is listed. n-3 DPA, lipids are derived from DPA*.

### Lipoxins and Lipoxin Pathway Markers

**Figure 3:**
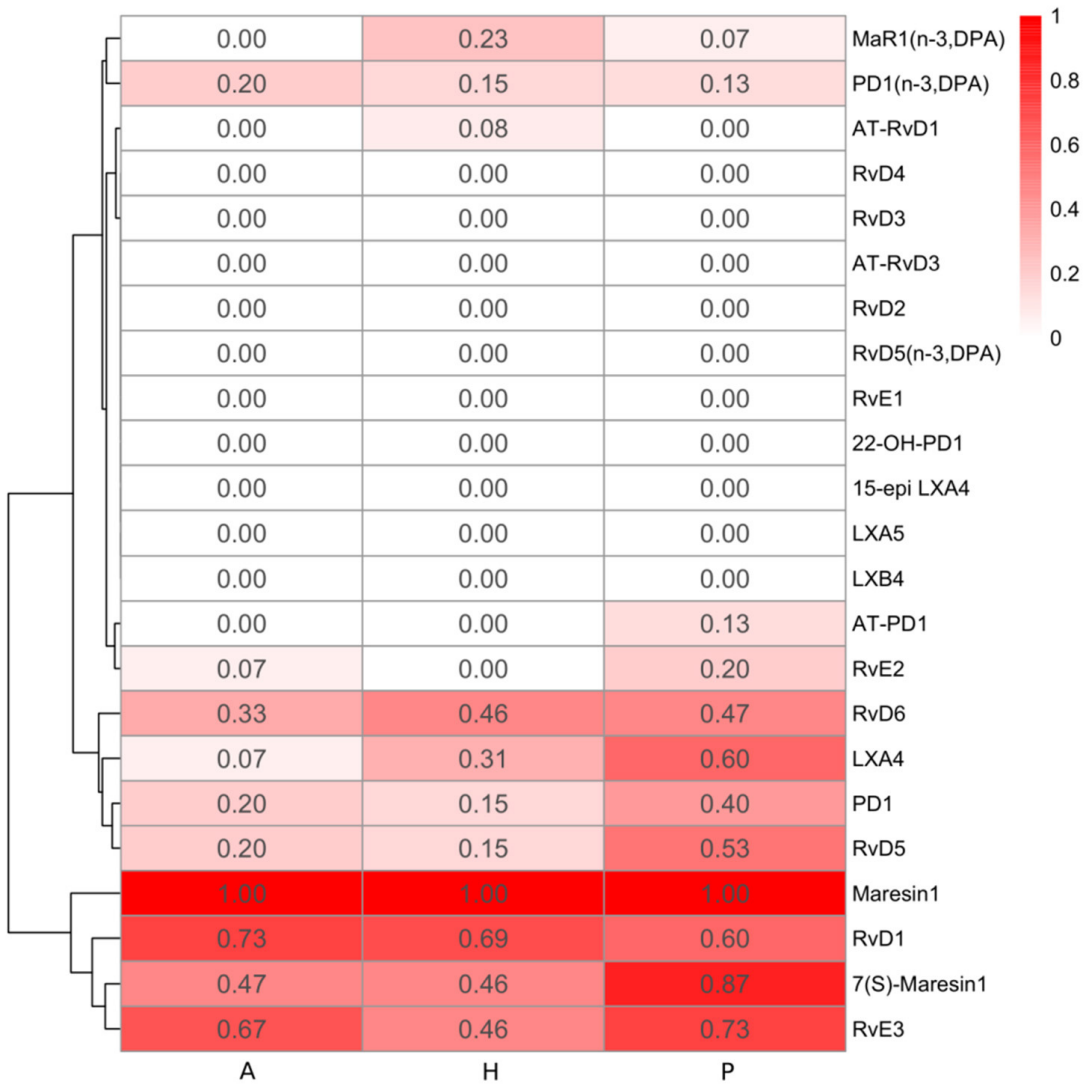
Heatmap of specialized pro-resolving lipid mediator (SPM) detection frequency. The detection frequency is calculated based on the number of samples where the specific SPM is detected divided by the total number of samples in each group (A: periodontitis after non-surgical therapy; H: healthy; P: periodontitis before non-surgical therapy). The color intensity reflects the calculated detection frequency.

Lipoxins are derived from omega-6 arachidonic acid. LXA4 was the only lipoxin detected in gingiva and was more commonly seen in the periodontitis prior to SRP group (9 out of the 15 total samples) than in the other two groups (Figure 3). All samples in the three groups had several detectable lipoxin pathway markers (5-HETE, 12-HETE, 15-HETE, 5(S),12(S)-DiHETE, and 12(S)-HHTrE). However, only levels of two pathway markers, 5-HETE and 15-HETE, were significantly higher in the periodontitis prior to SRP group compared to the periodontitis after SRP group (*p*-value = 0.01, 0.03, respectively) (Table 3).

### Resolvins and Resolvin Pathway Markers

Resolvins include E-series resolvins derived from omega-3 EPA and D-series resolvins derived from omega-3 DHA or DPA. RvE2 was only detected in few gingival samples of periodontitis subjects (3 samples in the P group and 1 sample in the A group) (Figure 3). Compared to RvE2, RvE3 was detected in more samples in the three groups. The E-series resolvin pathway markers (5-, 11-, 12-, 15(S)-, and 18-HEPE) were present in the majority of samples in the three groups, but only 15(S)-HEPE in the periodontitis prior to SRP group had a significantly higher level than in the periodontitis after SRP group (*p*-value = 0.03).

D-series resolvins, RvD1, RvD5, and RvD6, were detected in several gingival samples of the three groups. RvD1 was the most commonly detected, but not present in every sample (Table 3). The D-series resolvin pathway markers (4-, 7-, and 13-HDHA) were detected in the majority of, but not all, gingival samples. There was no significant difference in levels of the pathway markers between health and disease, but there was a significantly higher level of 4-, and 7-HDHA in the periodontitis prior to SRP group compared to the periodontitis after SRP group (*p*-value = 0.04, 0.03, respectively) (Table 3).

Generally, there was no significant difference in the levels of the E-series and D-series resolvins between the three groups. However, among the detected resolvins including RvE2, RvE3, RvD1, RvD5, and RvD6, four of them had the highest detection frequency in the periodontitis prior to SRP group (Figure 3).

### Protectins and Protectin Pathway Markers

Protectins are derived from omega-3 DHA or DPA. Protectin1 (PD1) was detected in a few samples of the three groups, with the highest detection frequency in the periodontitis prior to SRP group (Figure 3). There was no significant difference in levels of PD1 between groups. A protectin pathway marker, 17-HDHA, was detected in every gingival sample, and there was a significantly higher level of 17-HDHA in the periodontitis prior to SRP group compared to the after SRP group (*p*-value = 0.03) (Table 3).

### Maresins and Maresin Pathway Markers

Maresins were derived from omega-3 DHA or DPA. MaR1 and its pathway marker, 14-HDHA, were consistently present in every gingival sample in the three groups. There was no significant difference in the levels of maresins or 14-HDHA between groups (Table 3). 7(S)-Maresin 1 was the most frequently detected in the periodontitis prior to SRP group.

### Pro-inflammatory Arachidonic Acid Derivatives

The pro-inflammatory mediators (LTB4, PGD2, PGE2, PGF2a) were identified in a majority of the gingival samples. However, there was no significant difference in levels of any mediators between the three groups.

### Increased Levels of SPM Pathway Markers in Periodontitis Prior to Treatment

Among thirty-six detected lipid mediators, six lipid mediators had significantly higher levels in periodontitis prior to treatment than in periodontitis after treatment. These lipid mediators are pathway markers for different SPMs: 5-HETE and 15-HETE are involved in synthetic pathways of lipoxins; 15(S)-HEPE is involved in the synthetic pathway of E-series resolvins; 4-HDHA and 7-HDHA are involved in synthetic pathways of D-series resolvins; 17-HDHA is involved in synthetic pathways of protectins and D-series resolvins. These significant differences in pathway marker levels indicated pro-resolving activities are reduced following non-surgical periodontal treatment.

### Expression of SPM Receptor Genes in Gingival Tissues

Receptor gene expression profiles were distinct in patients with different conditions of periodontal inflammation (Figure 2). While evaluating individual receptor gene expression, all 7 receptor genes (*ALX, BLT1, ChemR23, GPR18, GPR32, GPR37*, and *LGR6)* were expressed in every gingival sample (Table 4). The expression of *BLT1* gene was significantly higher in the healthy group compared to the periodontitis prior to SRP and after SRP groups (*p*-value < 0.01). Furthermore, the expression of *GPR18* gene was shown to be significantly higher in the periodontitis prior to SRP group compared to the periodontitis after SRP group (*p*-value = 0.04), while the expression of *GPR32* gene showed the opposite pattern (*p*-value = 0.04). No other significant differences were seen in receptor gene expression between groups. Increased expression of specific receptor genes in different groups indicates that their corresponding SPMs can be involved in these clinical conditions to control inflammation in gingiva.

**Table 4:** Expression of SPM receptor genes in gingival tissues.

**SPM receptor gene**	**Relative expression in healthy (H)**	**Relative expression in Periodontitis before non-surgical therapy (P)**	**Relative expression in periodontitis after non-surgical therapy (A)**	**Relative fold change between H and P**	**Relative fold change between H and A**	**Relative fold change between A and P**
*ALX (FPR2)*	1.936	2.698	1.988	0.762	0.052	0.711
*BLT1 (LTB4R)*	**1.046**	**0.545**	**0.618**	**−0.502**	**−0.428**	−0.074
*ChemR23 (ERV1)*	2.157	1.642	1.79	−0.516	−0.367	−0.148
*GPR18 (DRV2)*	1.868	**1.98**	**1.295**	0.112	−0.572	**0.684**
*GPR32 (DRV1)*	0.563	**0.431**	**0.735**	−0.131	0.172	**−0.304**
*GPR37*	0.687	0.627	0.624	−0.059	−0.062	0.003
*LGR6*	0.919	0.808	0.740	−0.112	−0.179	0.067

*Numbers highlighted in bold indicate statistically significant difference (FDR adjusted p-value < 0.05). Expression levels are present in log_2_ mean of relative gene expression (2^−ΔΔCT^). The relative fold change is present in the log_2_ value between the two groups with the first group as the reference group*.

### Effects of Covariables on Lipid Levels and Gene Expression

Linear mixed-effects regression showed that maresin1, 7(S)-maresin1, 5(S),12(S)-DiHETE and LTB4 were associated with age (*p*-value = 0.043, 0.004, 0.093, 0.002, respectively) and RvD1 was associated with both age and BOP (*p*-value = 0.097, 0.075, respectively). After adjusting age, levels of maresin1, 7(S)-maresin1, 5(S),12(S)-DiHETE, and LTB4 were not significantly different between groups (*p*-value > 0.05). Levels of RvD1 were not significantly different between groups either while adjusting both age and BOP (*p*-value > 0.05).

For receptor gene expression, none of the genes was associated with age (*p*-value > 0.1) and there was no need for adjustment. With respect to the BOP effect, *GPR32* expression was associated with presence of BOP (*p*-value = 0.039). After adjusting for BOP, expression of *GPR32* gene in the periodontitis prior to treatment was significantly lower than in the periodontitis after treatment (*p*-value = 0.046) which was shown in the results of Wilcoxon signed rank test. Generally, age and BOP did not affect the results analyzed by Wilcoxon tests.

## Discussion

In this study, we report that the lipid mediator profiles were different between healthy, periodontitis and treated periodontitis in gingiva, based upon the distinct clustering of samples in PCA plots and results of ANOSIM. These results indicate lipid mediator profiles are associated with the state of periodontal inflammation. Additionally, there was a trend for higher detection frequency of these SPMs (out of thirteen detected SPMs, ten had the highest detection frequency in the periodontitis prior to SRP group) and increased levels of SPM pathway markers in periodontitis prior to treatment, which may give insight into the increased activity of SPM synthesis in periodontitis. Although subjects with periodontitis attempt to produce more SPMs to resolve inflammation, the levels might be still insufficient. As a result, chronic inflammation persists due to inefficient resolution of inflammation.

Specifically, results of LM-SPM metabololipidomics showed that six pathway markers, 5-HETE, 15-HETE, 15(S)-HEPE, 4-HDHA, 7-HDHA, and 17-HDHA, in periodontitis subjects prior to non-surgical therapy were significantly higher than in periodontitis subjects after non-surgical therapy. These lipid mediators involved in different SPM synthetic pathways represent specific characteristics in inflammation and resolution of inflammation.

Lipoxins are the only SPM family members derived from omega-6 fatty acids. The native molecule and its analogs have been used to effectively prevent periodontal tissue destruction in preclinical models (21). The increased level of 15-HETE, a pathway marker for lipoxins, suggests the activity of the lipoxin pathway might be more pronounced in inflamed gingival tissues prior to SRP. Although the levels of LXA4 were not significantly different between groups, LXA4 was the most frequently detected in patients with periodontitis before treatment compared to after treatment and healthy controls. These findings also confirm increased activity of lipoxin pathway in periodontitis prior to treatment (15). Conversely, the increased level of 5-HETE, which is a pathway marker for both leukotrienes and lipoxins indicates that the pro-inflammatory activity might also be higher in periodontitis subjects prior to SRP. LTB_4_, a potent chemoattractant for neutrophils into inflamed tissues (34), is the only frequently detected leukotriene in our samples and its levels were not significantly different between the three groups. A prior study demonstrated that levels of 15-HETE and 5-HETE increased in saliva and whole blood samples in patients with aggressive periodontitis compared to healthy controls (8). These results suggest both elevated omega-6-driven pro-resolving and pro-inflammatory activities in periodontitis might cause the imbalance in inflammation in periodontitis. This imbalance is reduced following non-surgical treatment.

Resolvins are the most studied SPMs in periodontal diseases. Prior work has shown that resolvins reduce inflammation and regenerate lost bone in experimental periodontitis preclinical models by inhibiting neutrophil infiltration, enhancing macrophage nonphlogistic phagocytosis and inhibiting osteoclast differentiation (20, 21). The level of 15(S)-HEPE, a pathway marker for E-series resolvins, was shown to be significantly higher in periodontitis prior to SRP than after SRP. RvE3 was the only E-series resolvin detected in gingiva of all three groups. The mean level and detection frequency of RvE3 in periodontitis subjects were higher than in healthy subjects, albeit, not statistically significant. Levels of 4-HDHA and 7-HDHA, the pathway markers for D-series resolvins, were significantly higher in periodontitis subjects prior to SRP than after SRP. Several D-series resolvins (RvD1, RvD5, RvD6) were detected in gingiva with no significantly different levels between the three groups. RvD5 and RvD6 had the highest detection frequency in periodontitis prior to treatment. These results show pronounced activity of resolvin pathways in gingiva of periodontitis subjects prior to non-surgical periodontal therapy.

Protectins regulate cellular activities similarly to other SPMs. PD1, also known as neuroprotectin D1, while produced in neural systems, has potent protective actions in retina, brain, and pain (35). Levels of PD1 were not significantly different between the three groups. The pathway marker for protectins and D-series resolvins, 17-HDHA, was identified in every gingival sample and had a significantly higher level in periodontitis prior to treatment than after treatment. Prior studies have shown that 17-HDHA regulates immune responses to control influenza virus infection and resolve inflammation in experimental colitis (36, 37). The increased level of 17-HDHA in periodontitis not only suggests pronounced activities of protectin and D-series resolvin pathways, but also indicates tissues may attempt to produce 17-HDHA to control periodontal inflammation.

MaR1 is important in stimulating macrophage phenotype switch from M1 to M2 macrophages, as well as inhibiting LTB4 formation (38). Also, MaR1 can enhance phagocytosis and bacterial killing of compromised macrophages and neutrophils collected from localized aggressive periodontitis patients (18). Levels of MaR1, and its pathway marker, 14-HDHA, were not significantly different between groups. However, MaR1 was the only detected SPM present in every gingival sample. This suggests that the stability of MaR1 might be better than other SPMs in gingival tissues.

Results of metabololipidomics showed that none of SPMs had significantly different levels between the three groups. Although SPMs had been detected in many human specimens, the limited quantity of SPMs might not be easily detected in one small piece of gingival tissue collected between two teeth. Pathway markers usually have higher levels than SPMs (8, 9, 39) given they are generally less physiologically active than SPMs. This is likely why there were no significant differences in SPM levels, but levels of several pathway markers were significantly different between the periodontitis prior to and after treatment in the gingival tissues. The stability of these pathway markers makes them more reliable quantitative indices than SPMs.

Although presence of SPMs is paramount to resolving inflammation, if their corresponding receptors are absent from key cells in tissues, their potential actions would essentially become void. In pre-clinical models, deficient expression of the SPM corresponding receptors is associated with increased inflammation (29) and overexpression of the corresponding receptors is associated with resolution of inflammation (40). The present study showed all seven tested SPM receptor genes were expressed in the gingival tissues. Five out of seven receptor genes had lower mean expression levels in periodontitis subjects prior to treatment compared to healthy and/or periodontitis subjects after treatment. Along with the increased levels of SPM pathway markers and a higher detection frequency of SPMs in periodontitis before treatment, the deficient receptor gene expression in periodontitis suggests that these subjects, in an attempt to resolve the excess inflammation, may produce more SPMs, but the deficiency in SPM receptor expression on cells renders the actions of SPM ineffective. As a result, the SPMs are not efficiently used, and inflammation persists.

SPM receptors have different roles in inflammation depending on their corresponding SPMs. Expression of *BLT1* gene was shown to be significantly increased in health compared to periodontitis before and after treatments suggesting that, in the healthy state, its corresponding SPMs, E-series resolvins, can be efficiently used to resolve inflammation (41). It was found that expression of *GPR18 (DRV2)* gene was significantly higher in periodontitis subjects prior to nonsurgical therapy, while expression of *GPR32 (DRV1)* gene was significantly increased after nonsurgical therapy (42). The corresponding SPM for GPR18 (DRV2) receptor was RvD2 and the corresponding SPMs for GPR32 receptors were RvD1, RvD3, and RvD5. Studies have shown that increased GPR18 or GPR32 receptor expression on macrophages significantly increases D-series resolvin induced phagocytosis of bacteria and apoptotic neutrophils (29, 43). Elevated expression of the SPM receptor is helpful for inducing resolution of inflammation. It is possible that the significantly increased expression of *GPR18* gene and the increased levels of the D-series resolvin pathway markers (4-HDHA, 7-HDHA, 17-HDHA) in periodontitis indicates the attempt of the host response to resolve inflammation via the RvD2-GPR18 resolution axis in periodontal disease. Still, these subjects may require considerably higher levels of expression of both RvD2 and GPR18 to effectively resolve periodontal inflammation. Moreover, these findings also demonstrate the potential to increase RvD2 levels through the external application of RvD2 or taking polyunsaturated fatty acids (PUFA) supplements (44, 45) to aid in resolution given the expression of GPR18 receptor could be elevated in gingival tissues of periodontitis subjects. The significantly increased expression of *GPR32* gene after non-surgical therapy also suggests the role of D-series resolvins in resolution of inflammation in periodontitis. Overall, as all tested SPM receptor genes were present in both healthy and diseased gingival samples, any SPM may potentially be used in an external application to help in the resolution of periodontal disease.

Levels of SPMs and relevant lipid mediators were identified in oral cavity. Elabdeen et al. measured levels of SPMs and lipid mediators in gingival crevicular fluid (GCF), saliva, and serum samples in subjects with aggressive periodontitis using mass spectrometry (8). In comparison, the current study measured levels of these lipid mediators in gingival tissue samples in moderate to severe chronic periodontitis, which is now referred to as stage II or III periodontitis (46). In Elabdeen et al., they also found that several pathway markers, such as 5-HETE, 15-HETE, 15(S)-HEPE, and 17-HDHA, were significantly higher in the GCF, saliva or serum of aggressive periodontitis subjects compared to healthy controls (8). In this current study, the levels of several pathway markers were significantly higher in the gingiva of periodontitis subjects prior to non-surgical therapy compared to subjects with periodontitis after non-surgical therapy, but not in healthy subjects. It is possible that the younger age and specific disease phenotype of subjects with aggressive periodontitis in Elabdeen et al. caused different results compared to our subjects with periodontitis. Importantly, these lipid mediators were measured in different types of samples. In a recently published study (22), significantly decreased LXA4 and increased PD1 as well as MaR1 salivary levels measured by ELISA were detected in periodontitis subjects in comparison to healthy controls. These results were not seen in the current study and the Elabdeen et al. study (8). These three studies had different samples (GCF, saliva, serum vs. gingiva), analytical methods and patient groups, which make direct comparison challenging.

The current study found no significant differences in pro-inflammatory lipid mediator levels, including LTB4, PGD2, PGE_2_, and PGF2a, in any of the three groups of the gingival samples. These results were not consistent with previous findings showing increased levels of pro-inflammatory mediators in the gingival crevicular fluid (47, 48) or gingival tissues (49) of periodontitis subjects. It is possible that the levels of these fast-acting pro-inflammatory lipid mediators are very dynamic in the tissues. Although these lipid mediators may be expressed in short-term gingival inflammation, they cannot reliably signify the disease status. Alternatively, the sample size in the current study was too small to detect statistical significance in the levels of these mediators.

Demographics analysis revealed that there was a significant difference in age between the healthy and periodontitis subjects. The subjects without periodontitis were younger than the periodontitis subjects, which reflects the well-known fact that age is associated with severity of periodontal disease (50, 51). Several of the healthy subjects in the study potentially had gingivitis rather than gingival health (52). In clinical settings, it is challenging to find subjects with very few BOP sites. Analyzing samples collected from gingivitis subjects potentially can explain why gingival inflammation present in these subjects does not progress to periodontitis. In the current study, the impact of age and presence of BOP did not impact the final results.

It is well known that the oral microbiota shifts following periodontal treatment, given that biofilm is removed and inflammation is reduced (53). Interactions between immune cells and bacterial species are prominent in periodontitis (54). Preclinical studies also show that shifts in the oral microbiota are associated with the resolution of inflammation induced by SPM in experimental periodontitis (20). Therefore, in the current study, changes of lipid mediator level and receptor gene expression might be correlated with changes of microbiota following periodontal treatment. Further studies are needed to investigate the associations between changes in lipid mediator and receptor gene expression profiles and changes in the oral microbiome profiles in different inflammatory conditions.

The limitation of the current study is that the expression of receptors at the protein level was not directly measured, since protein levels and mRNA levels are not always highly correlated (55). Additionally, only six out of thirty-six detected lipid mediators had significantly different levels between periodontitis prior to treatment and after treatment. Non-significant differences in other lipid mediator levels might be due to lack of biological relevance or the limited sample size. Periodontitis subjects in the study only received non-surgical therapy. The clinical outcomes, such as probing depth reduction, were not as profound as outcomes following surgical therapy (56). However, distinct lipid mediator profiles and receptor gene expression profiles were still associated with significant changes of inflammatory conditions following non-surgical therapy. It should be noted that the clustering patterns in lipid mediator profiles and receptor gene expression profiles are not the same. The return of the receptor gene expression profile following treatment to healthy status was not seen in lipid mediator profile following treatment. Also, the difference in receptor gene expression profiles between non-treated periodontitis and healthy controls were more significant than the difference in lipid mediator profiles.

In conclusion, for the first time, levels of SPMs and relevant lipid mediators and expression of SPM receptor genes were measured in human gingiva collected from subjects with or without periodontitis. Lipid mediator and receptor gene expression profiles were associated with the statuses of periodontal inflammation. Higher levels of several SPM pathway markers were present prior to SRP compared to after SRP in gingiva indicating inflammation-induced pro-resolution activity, but SPM corresponding receptors appeared to be deficient in periodontitis. Elucidation of these lipid mediator and receptor gene expression profiles allows for better understanding of the role that SPMs have in periodontitis, which in turn may assist in designing new screening techniques or treatment regimens for the resolution of periodontitis.

## Data Availability Statement

The datasets generated for this study are available on request to the corresponding author.

## Ethics Statement

The studies involving human participants were reviewed and approved by University of Texas Health Science Center at Houston (UTHealth) Committee for the Protection of Human Subjects. The patients/participants provided their written informed consent to participate in this study.

## Author Contributions

TV and C-TL contributed conception and design of the study. BF and C-TL collected clinical samples. BF, NB, and C-TL processed clinical samples and performed experiments. BF, NB, KM, LZ, WC, WZ, and C-TL performed the data analysis. KM, SA, RW, NA, TV, and C-TL contributed to data interpretation. BF and C-TL wrote the first draft of the manuscript. All authors contributed to the article and approved the submitted version.

## Conflict of Interest

The authors declare that the research was conducted in the absence of any commercial or financial relationships that could be construed as a potential conflict of interest.

## Abbreviations

10S,17S-DiHDHA: 10S,17S-dihydroxydocosahexaenoic acid
5(S),6(R)-DiHETE: 5(S),6(R)-dihydroxyeicosatetraenoic acid
5(S),12(S)-DiHETE: 5(S),12(S)-dihydroxyeicosatetraenoic acid
5(S),15(S)-DiHETE: 5(S),15(S)-dihydroxyeicosatetraenoic acid
5(S),15(S)-DiHEPE: 5(S),15(S)-dihydroxyeicosapentaenoic acid
4-HDHA: 4-hydroxydocosahexaenoic acid
7-HDHA: 7-hydroxydocosahexaenoic acid
13-HDHA: 13-hydroxydocosahexaenoic acid
14-HDHA: 14-hydroxydocosahexaenoic acid
17-HDHA: 17-hydroxydocosahexaenoic acid
5-HEPE: 5-hydroxyeicosapentaenoic acid
11-HEPE: 11-hydroxyeicosapentaenoic acid
12-HEPE: 12-hydroxyeicosapentaenoic acid
15(S)-HEPE: 15(S)-hydroxyeicosapentaenoic acid
18-HEPE: 18-hydroxyeicosapentaenoic acid
5-HETE: 5-hydroxyeicosatetraenoic acid
11-HETE: 11-hydroxyeicosatetraenoic acid
12-HETE: 12-hydroxyeicosatetraenoic acid
15-HETE: 12-hydroxyeicosatetraenoic acid
12(S)-HHTrE: 12(S)-hydroxyheptadecatrienoic acid
DHA: docosahexaenoic acid
DPA: docosapentaenoic acid
EPA: eicosapentaenoic acid
LMs: lipid mediators
LTB4: leukotriene B4
20-OH-LTB4: 20-hydroxy-leukotriene B4
20-COOH LTB4: 20-carboxy-leukotriene B4
LxA4: lipoxin A4
15-epi LxA4: 15-epi lipoxin A4
LxA5: lipoxin A5
LxB4: lipoxin B4
MaR1: maresin 1
Maresin 1 (n-3, DPA): maresin 1 (derived from docosapentaenoic acid)
7(S)-MaR1: 7-epi maresin 1
PGD2: prostaglandin D2
PGE2: prostaglandin E2
PGF2a: prostaglandin F2a
PD1: protectin D1
PD1 (n-3, DPA): protectin D1 (derived from docosapentaenoic acid)
AT-PD1: aspirin-triggered protectin D1
22-OH-PD1: 22-hydroxy-protectin D1
RvD1: resolvin D1
AT-RvD1: aspirin-triggered resolvin D1
RvD2: resolvin D2
RvD3: resolvin D2
AT-RvD3: aspirin-triggered resolvin D3
RvD4: resolvin D4
RvD5: resolvin D5
RvD5(n-3, DPA): resolvin D5 (derived from docosapentaenoic acid)
RvD6: resolvin D6
RvE1: resolvin E1
RvE2: resolvin E2
RvE3: resolvin E3
SPMs: specialized pro-resolving lipid mediators
TxB2: thromboxane B2.
